# Investigating the Effects of pH and Temperature on the Properties of Lysozyme–Polyacrylic Acid Complexes via Molecular Simulations

**DOI:** 10.1021/acsomega.5c03767

**Published:** 2025-07-31

**Authors:** Sisem Ektirici, Vagelis Harmandaris, Anastassia N. Rissanou

**Affiliations:** † Computation-Based Science and Technology Research Center, 338376 The Cyprus Institute, Nicosia 2121, Cyprus; ‡ Department of Mathematics and Applied Mathematics, 37777 University of Crete, GR-71409 Heraklion, Greece; § Institute of Applied and Computational Mathematics, 54570 Foundation for Research and Technology Hellas, IACM/FORTH, GR-71110 Heraklion, Greece; ∥ Theoretical and Physical Chemistry Institute, National Hellenic Research Foundation, 48 Vassileos, Constantinou Avenue, GR-11635 Athens, Greece

## Abstract

Studying protein–polymer complexes at the molecular level is crucial for understanding how polymers interact with proteins and affect their stability and function. The complexation process of lysozyme (LYZ) and poly­(acrylic acid) (PAA) is highly dependent on pH and temperature, influencing both the stability and binding dynamics of the interaction network. Using atomistic molecular dynamics simulations, we explored how these environmental factors shape the binding strength, molecular rearrangements, and conformational adaptability of the [LYZ–PAA] complexes. The results reveal that pH has a pronounced effect on the resulting complexes, where higher pH disrupts protein–polymer interactions due to increased electrostatic repulsion. At the same time, an increase in temperature leads to more transient and fluctuating interactions while maintaining overall binding stability. Structural analysis further supports these trends, showing that higher temperatures promote flexibility, while higher pH leads to greater conformational expansion and reduced stability. Through association rate calculations and hydrogen bonding analysis, we identified key residues, such as arginine and lysine, that dominate the LYZ/PAA interaction at lower pH levels, while higher pH values promote a shift toward hydrophobic interactions. Our findings highlight the critical role of pH and temperature in controlling molecular interactions, offering valuable insights for applications in biomaterials and protein-based delivery systems.

## Graphical abstract

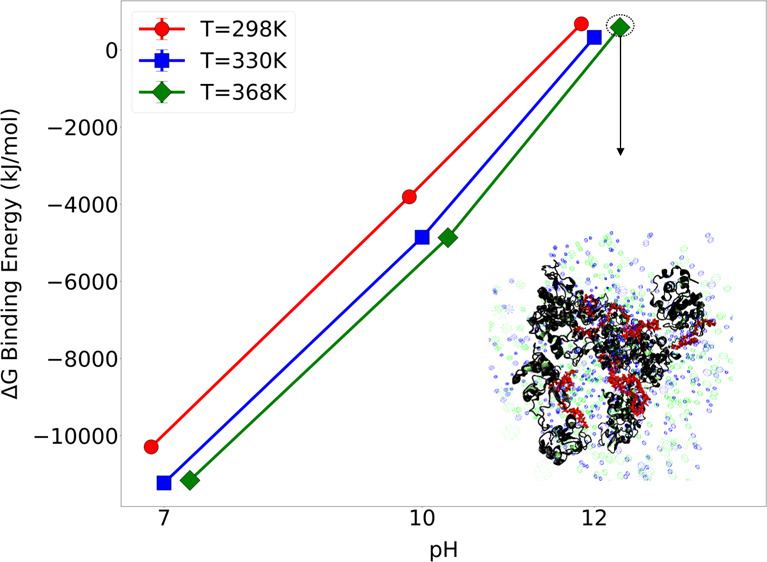

## 1 Introduction

Proteins and polyelectrolytes play a fundamental role in various biological and industrial systems, forming complexes that drive key physiological functions^1^ and technological applications.^2,3^ The association between proteins and synthetic or biological polyelectrolytes is a complex and dynamic process influenced by multiple factors, including electrostatic interactions, hydrogen bonding, hydrophobic effects, and solvent-mediated forces. Among these factors, pH and temperature are particularly critical, as they directly affect the charge distribution, the conformational stability, and the binding affinity of proteins and polyelectrolytes.^2,3^ Understanding how these environmental conditions govern protein–polyelectrolyte interactions is essential for applications ranging from biomedical engineering^4,5^ and drug delivery^6,7^ to food science^8–10^ and biomaterials development.^11,12^

pH particularly plays a pivotal role in protein-polyelectrolyte complexation, as it determines the protonation state of amino acid residues, thereby modulating electrostatic interactions between the two components. Proteins contain both positively and negatively charged residues, and their net charge depends on the pH relative to their isoelectric point.^13^ Changes in charge distribution, as a result of pH, influence the protein–polyelectrolyte complexation, particularly the stability of the formed network and the binding affinity. Consequently, controlling pH conditions is a key strategy in optimizing protein–polymer formulations for biomaterials, biosensors, and drug encapsulation systems. Murmiliuk et al. demonstrated that protein–polyelectrolyte complexation is highly pH-dependent, with positively charged polyelectrolytes assembling with insulin, above its isoelectric point, due to electrostatic attraction, while disassembly occurs below the isoelectric point due to charge repulsion.^14^ Balabushevich et al. developed a novel microencapsulation technique for protein-containing aggregates by applying polyelectrolyte multilayers, using dextran sulfate and chitosan in a layer-by-layer adsorption process. Their findings demonstrated that the resulting micro particles remained stable at pH values below 5, while protein release was triggered at pH levels similar to that of the small intestine.^15^

Similarly, temperature is a crucial factor in protein–polyelectrolyte interactions, as it affects molecular motion, solvent dynamics, and interaction (free) energy landscapes. Increasing the temperature generally enhances thermal fluctuations, which can either stabilize or destabilize protein–polymer interactions depending on the balance between entropic and enthalpic contributions. At high temperatures, excessive thermal agitation may lead to disruptions in hydrogen bonding networks and structural unfolding of proteins. Understanding the temperature-dependent behavior of protein-polyelectrolyte complexes is particularly important in biopharmaceutical formulations,^16,17^ enzyme stabilization,^18,19^ and industrial polymer–protein processing,^20,21^ where precise temperature control is necessary to maintain functional integrity and interaction stability. Bukala et al. conducted a thermodynamic investigation of protein–polyelectrolyte interactions, showing that the binding affinity of cationic polyelectrolytes to bovine serum albumin (BSA) is influenced by temperature. Using isothermal titration calorimetry (ITC), they demonstrated that temperature variations (20–37 °C) impact the free energy of binding, with contributions arising from the release of counterions from the polymer and the release of bound water molecules.^22^ Yu et al. demonstrated that the binding affinity between human serum albumin (HSA) and poly­(acrylic acid) (PAA) increases with temperature, primarily due to entropy-driven counterion release. Their isothermal titration calorimetry experiments and coarse-grained simulations confirmed that PAA binds at the Sudlow II site of HSA, with temperature playing a key role in modulating the strength and stability of the interaction.^23^

MD approaches have been successfully applied to study DNA–polycation interactions, revealing that linear poly l-lysine (PLL) binds DNA in a concerted manner, while grafted oligolysines interact independently, resulting in distinct structural and thermodynamic behaviors. Additionally, simulations demonstrated that the presence of a hydrophobic backbone in grafted oligolysines weakens their binding to DNA compared to linear PLL, leading to a nonmonotonic variation in binding free energy, which correlates with experimentally observed transfection efficiencies.^24^ Furthermore, MD simulations have been widely used to analyze the binding patterns of synthetic cationic polymers with biomolecules, such as DNA, revealing that polyethylenimine (PEI), poly l-lysine (PLL), and polyallylamine stabilize DNA complexes through electrostatic interactions, while polyvinylamine (PVA) adopts a unique binding mode by embedding into the DNA major groove.^25^ This difference in binding behavior suggests that structural and electrostatic factors govern the stability and adaptability of protein-polyelectrolyte complexes in aqueous environments. Moreover, computational studies on LYZ–polyelectrolyte interactions have shown that binding free energy (Δ*G*_binding_) can be decomposed into contributions from counterion release and direct electrostatic interactions, as demonstrated in the interaction of LYZ with dendritic polyglycerolsulfate (dPGS). In this system,^26^ MD simulations and experimental data revealed a strong enthalpy–entropy compensation effect, where binding enthalpy is counterbalanced by solvation effects, rather than purely electrostatic interactions, which stands in the case of protein–DNA binding.^24^

Although protein–polyelectrolyte systems are frequently studied in the literature,^27–29^ there has been little work on the combined effects of pH and temperature on the interactions and structural properties of these systems. Joshi et al. developed injectable “smart” microspheres sensitive to both temperature and pH, showing sustained protein release under ischemic conditions, contrasting with rapid clearance at physiological pH.^30^ Jin et al. enhanced thrombolytic therapy with pH/temperature dual-responsive protein–polymer conjugates, demonstrating masked uPA bioactivity at normothermic pH and its recovery at hypothermic, acidic conditions.^31^

In a recent work, lysozyme–PAA ([LYZ–PAA]) systems in water, under physiological conditions (pH 7), revealed temperature-induced conformational changes and structural shifts. Mechanisms behind aggregation were elucidated, suggesting new bond formations, after a specific thermal treatment, which causes structural and energetic changes, enhancing complex stability.^32^ Building upon this foundation, the current study aims to expand our understanding of protein–polyelectrolyte complexation by systematically exploring the combined effects of pH and temperature on the binding and stability of [LYZ–PAA] complexes in multimolecule systems, considering additional factors that influence intermolecular interactions, structural adaptation, and complexation pathways. LYZ was selected for this study due to its well-characterized structure and its relevance in protein–polyelectrolyte research.^33–35^ As a positively charged globular protein at a neutral pH, LYZ serves as an ideal model system for studying electrostatic-driven interactions with polyanionic polymers. Moreover, its high thermal stability allows for the investigation of temperature-dependent effects on complexation without significant loss of structural integrity. PAA is a biocompatible macromolecule and was chosen as the polyelectrolyte component due to its negatively charged carboxyl groups, which provide a strong electrostatic complement to LYZ’s positively charged residues. PAA is widely used in biomedical applications,^36,37^ coatings^38^ and controlled drug release,^39–41^ making its interaction with proteins a subject of significant interest in both fundamental and applied research.

Understanding how pH and temperature modulate the molecular binding and structural dynamics of [LYZ–PAA] complexes offers direct implications for controlled drug delivery applications. Both PAA and LYZ are used in biomedical contexts—including drug encapsulation^42,43^ and targeted release platforms.^44–46^ Our findings provide molecular-level insight into how environmental conditions can be harnessed to design stimuli-responsive delivery systems, revealing the mechanisms governing the assembly and stability of LYZ–PAA networks. This contributes to a deeper understanding of the specific conditions that promote or disrupt network formation. Identifying these conditions and the underlying interactions that drive network formation can ultimately inform strategies for tuning the encapsulation efficiency, release behavior, and overall performance of the delivery system.

To capture the molecular details of LYZ–PAA interactions under varying pH and temperature values, we perform atomistic MD simulations of LYZ–PAA, exploring the formation process and the stability of the [LYZ–PAA] complex under different environmental conditions. The goal of this work is to investigate the binding and the stability of [LYZ–PAA] complexes formed by multiple LYZ and PAA molecules. For this, we examine how hydrogen bonding, binding free energy, structural rearrangements, and molecular contact dynamics respond to environmental changes, resolving molecular interactions of biomolecular complexation. By systematically evaluating the effects of pH and temperature on LYZ–PAA complexation, this study aims to provide key insights into the molecular mechanisms that govern protein–polyelectrolyte interactions.

## 2 Model and Systems

### 2.1 Simulation Details

Protein–polymer mixtures were simulated in an aqueous environment under various pH and temperature conditions. Three pH values: 7, 10, 12, and three temperature values: 298, 330, and 368 K were utilized. pH 7 represents physiological conditions, while pH values of 10 and 12 were selected to investigate how the progressive deprotonation of LYZ affects its electrostatic interactions with the polymer and alters the stability of the complex. Similarly, 298 K represents ambient conditions, while 330 and 368 K were selected to examine how increasing temperature affects the structural stability of LYZ and its interactions with the polymer. The mass ratio, *M*_PAA_/*M*_LYZ_, in the mixture was adjusted to 0.1 by mixing 16 LYZ molecules and 8 PAA chains, each consisting of 40 monomer units. The mass ratio was chosen to maintain a physiologically relevant and computationally feasible system, where the effect of PAA on LYZ structure and dynamics can be systematically analyzed without introducing excessive polymer crowding. Protein water solutions, including 16 LYZ proteins, were also simulated as reference systems. The PAA chains were modeled assuming 50% deprotonation of carboxylic groups, based on a p*K*_a_ of 4.5,^47^ monomer spacing of 0.27 nm, and a Bjerrum length of 0.7 nm.^48^ Simulations of 300 ns were conducted for all of the systems. The initial configurations of LYZ at different pH levels were derived from the Protein Data Bank (PDB) code 1GWD, with protonation states adjusted for various pH conditions using the CHARMM-GUI server.^49^ Protonated residues at different pH levels are shown in Figure S1. Counter ions (Na^+^ and Cl^–^) were added in order to neutralize the system and to set the ionic strength of the solution to *I* = 0.15 M. Table 1 summarizes the simulated systems, showing the pH and temperature combinations and the number of atoms for each component.

**Table 1:** Summary of the Simulated Systems^a^

system	pH	*T* (K)	total number of atoms	number of water atoms	number of LYZ atoms	number of Na atoms	number of Cl atoms
LYZ	7	298	403,235	371,007	31,360	370	498
LYZ	10	298	403,065	371,361	30,928	372	404
LYZ	12	298	404,052	371,952	31,296	434	370
[LYZ–PAA]	7	298, 330, 368	403,211	368,343	31,360	402	370
[LYZ–PAA]	10	298, 330, 368	416,354	381,822	30,928	498	370
[LYZ–PAA]	12	298, 330, 368	403,282	368,286	31,296	594	370

a The table includes the system’s name, the pH value, the temperature (K), and the number of atoms of each component. The molecular composition in the mixture corresponds to 16 LYZ and 8 PAA molecules, whereas bulk LYZ contains 16 proteins.

All simulations were performed using the GROMACS simulation package (version 2023),^50^ employing the all-atom AMBER99SB-ILDN^51,52^ force field. The SPCE^53,54^ water model was used to represent water molecules in the MD simulations. Simulations were conducted by using the leapfrog integrator with a time step of 1 fs in the NPT statistical ensemble. Pressure coupling was performed using the Berendsen method,^55^ with isotropic scaling, time constant of 1.0 ps, and reference pressure of 1.0 bar. The temperature was controlled using the V-rescale thermostat,^56^ with separate coupling for the protein and nonprotein groups. Short-range electrostatic and van der Waals interactions were treated with cutoffs of 1.0 nm, while long-range electrostatics were calculated using the Particle Mesh Ewald (PME) method with cubic interpolation of order 4 and a Fourier grid spacing of 0.16 nm. Dispersion corrections were applied for both energy and pressure to account for the truncation effects. Periodic boundary conditions were applied in all directions.

### 2.2 Association Rate Calculation

The raw data from the GROMACS pairdist tool consisted of distances *d*_*i*,*j*,*k*_ for each residue *i*, frame *j*, and polymer chain *k*. For each of the 16 proteins, in order to isolate the closest contact of each residue to a polymer chain, within a given frame, we took the minimum distance across the eight polymer chains, denoting it as:

di,j(min)=mink∈{1,···,8}di,j,k 1

resulting in 16 × 129 values (16 proteins × 129 amino acids per protein) for each frame. We analyze *m* (here *m* = 10), uncorrelated frames after equilibrium, which correspond to about the last 100 ns of the trajectory. Then, we compute a single average distance per residue *d̅*_*i*_ over these frames for each protein molecule, through:

$${\overset{-}{d}}_{i}=\frac{1}{m}\sum_{j=1}^{m}d_{i,j}^{\left(\min\right)}$$ 2

Producing this average distance, we assume the time stability of the network formed between protein molecules and polymer chains. Here, *d̅*_*i*_ represents the protein-specific average distance of each residue *i*. Then, to further quantify how many of the residues are within a chosen threshold distance (set to 0.35 nm, which corresponds to the distance criterion for hydrogen bonding), we introduced an indicator function *I*_*i*_^*c*^ for each residue and protein, defined as

$$I_{i}^{c}=\{\begin{array}{cc} 1, & \mathrm{if}\;{\overset{-}{d}}_{i}\geq 0.35\;\mathrm{nm}\\ 0, & \mathrm{if}\;{\overset{-}{d}}_{i}\leq 0.35\;\mathrm{nm} \end{array}$$ 3

where, *c* is the protein index (*c* = 1–16). Next, we averaged the indicator values over all 16 protein molecules, for each residue, and expressed this as a percentage, according to eq 4, representing the residue’s association rate (AR) across proteins.

$$\mathrm{AR}(\%)=\left(\frac{\sum_{c=1}^{16}I_{i}^{c}}{16}\right)\times 100$$ 4

This calculation provides a measure of association rates by accounting for both time (averaging over frames) and protein molecules (averaging across 16 proteins). At the same time, for each time frame and for each protein molecule, we evaluate time-dependent variability and variability among protein molecules. Therefore, this algorithm effectively monitors the time stability of the formed network and the effect of the local environment around each residue. In this context, “environment” refers to the spatial arrangement of each residue relative to the polymer chains.

## 3 Results and Discussion

### 3.1 Effect of pH and Temperature on the [LYZ–PAA] Complexation

#### 3.1.1 Energetics

To rigorously assess the effects of pH and temperature on protein–polymer complexation, interaction energies and Gibbs free energies, along with their components, were quantified to provide a detailed thermodynamic perspective for the binding affinity. In addition, both inter- and intraspecies of hydrogen bonds were calculated to comprehensively describe the molecular interactions. Then, radial distribution functions, *g*(*r*), were utilized to analyze the distances within and between the simulated species, enhancing our understanding of their spatial relationships. Finally, the influence of pH and temperature on the polymer–protein approximation at the amino acid level was analyzed through association rate calculations.

The potential interaction energies between LYZ and PAA, calculated as the sum of the Lennard-Jones and short-range Coulomb energies, are presented in Table 2, averaged over the last 100 ns of the trajectory. It is observed that as pH increases, the total interaction energy also increases (negative values), suggesting a weakening of the attraction between the two molecules. However, the effect of temperature is nonmonotonic as well as not similar dependent on each pH value. Temperature increase from 298 to 368 K leads to an increase in total energy at all pH levels (i.e., attenuation of interactions). More specifically, at pH 7 and 10, the energies are within statistical uncertainties for the two lower temperature values (298 and 330 K). Since these values are strongly fluctuating, the temperature effect can be assigned only when *T* rises to 368 K, where a clearly stronger attraction is observed due to increased contacts (Figure 10) as a result of the enhanced thermal fluctuations. However, at pH 12, the net charge of LYZ is negative (−4), resulting in reduced interactions in the aqueous solution at lower temperatures, compared to the two lower pH levels. With the temperature rising to 330 K, the enhanced thermal fluctuations lead to a significant increase in interactions, since the increased molecular motion enhances structural flexibility, enabling closer approach and new transient contacts between the protein and the polymer. Further increase of temperature has no additional effect, and similar energy values are observed, within statistical uncertainties. Furthermore, in Figure S2, the time evolution of energy at different pH and temperature levels determines the equilibrium state of the systems, which in all cases is achieved beyond ∼200 ns.

**Table 2:** Lennard-Jones, Short-Range Coulombic, and Total Interaction Energies (kJ/mol) of the [LYZ–PAA] Complexes at Different pH and Temperature Conditions

system	condition	Lennard-Jones	SR-Coulombic	total
[LYZ–PAA]	pH = 7, *T* = 298 K	–2506.5 ± 95.5	–13183.4 ± 442.7	–15689.9 ± 463.3
[LYZ–PAA]	pH = 7, *T* = 330 K	–2730.6 ± 127.4	–12152.1 ± 445.9	–14882.74 ± 483.1
[LYZ–PAA]	pH = 7, *T* = 368 K	–3295.8 ± 118.8	–16453.1 ± 653.8	–19748.9 ± 674.4
[LYZ–PAA]	pH = 10, *T* = 298 K	–3190.0 ± 90.8	–9929.7 ± 277.9	–13119.8 ± 274.7
[LYZ–PAA]	pH = 10, *T* = 330 K	–2631.4 ± 112.8	–8635.8 ± 487.5	–11267.2 ± 539.3
[LYZ–PAA]	pH = 10, *T* = 368 K	–3722.4 ± 120.9	–12451.6 ± 541.5	–16174.0 ± 524.8
[LYZ–PAA]	pH = 12, *T* = 298 K	–1517.7 ± 116.0	–6351.9 ± 241.1	–7869.7 ± 251.8
[LYZ–PAA]	pH = 12, *T* = 330 K	–2337.1 ± 107.8	–9028.4 ± 445.9	–11365.5 ± 485
[LYZ–PAA]	pH = 12, *T* = 368 K	–2535.5 ± 116.4	–8665.1 ± 514.8	–11200.6 ± 541.3

While interaction energies provide insight into the immediate forces at play, Gibbs free energy of binding (Δ*G*_binding_) measures the stability and spontaneity of the protein–polymer complex, accounting for changes in enthalpy and entropy, to determine the thermodynamic favorability of the protein–polymer interactions under various conditions; i.e., the change in Gibbs free energy represents the change of the association energy during the formation of the complex. The calculation of Δ*G*_binding_ values using the MM-PBSA (Molecular Mechanics Poisson–Boltzmann Surface Area) method^57,58^ is described in SI. As observed in Figure 1 and Table S1, the Δ*G*_binding_ value increases with increasing pH, while changes in temperature have a milder impact.

**Figure 1:**
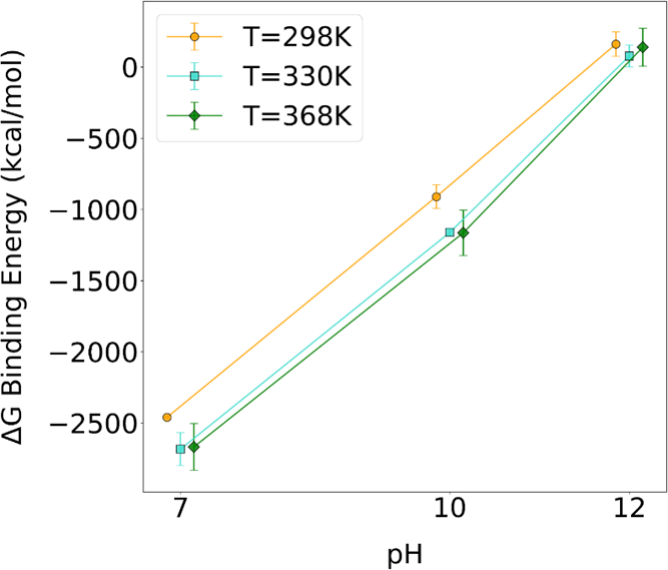
Comparison of Δ*G*_binding_ values for [LYZ–PAA] complexes at the equilibrium state across various pH and temperature values.

An increase in pH leads to a significant increase in Δ*G*_binding_ values, indicating a decrease in attractive interactions between the protein and polymer. The change in the charge distribution of the protein explains this, as shown in Figure S1. The LYZ protein’s overall positive charge decreases with increasing pH, becoming negative at pH 12. As the protein’s charge becomes increasingly negative, it has a weaker attraction to the PAA polymer, which also carries a total negative charge. This inference can be observed in the Δ*G*_binding_ components in Table S1 and Figure S3, where the Δ*E*_Coul_ value increases with increasing pH. It is noteworthy that at pH 12, these interactions become repulsive and the binding between LYZ and PAA comes as a result of the van der Waals attraction, hydrogen bonding (discussed in the following), and entropic contribution, illustrating the interactive effects of pH and temperature on the assembly process. The effect of temperature, on the other hand, shows that there is an obvious increase in the binding affinity when the temperature rises from 298 to 330 K, whereas the impact of temperature on Δ*G*_binding_ is minimal between 330 and 368 K. However, it is still possible to observe that increased temperature slightly enhances the attractive interactions between the two molecules. This trend is consistent with the work of Ran et al., who used isothermal titration calorimetry (ITC) to show that LYZ–polyelectrolyte binding is primarily entropy-driven due to counterion release, and that the Gibbs free energy remains nearly unchanged with increasing temperature, in agreement with our simulation results.^59^

#### 3.1.2 Hydrogen Bonds

To further elucidate the molecular interactions, we also performed a hydrogen bond analysis to investigate how variations in pH and temperature affect hydrogen bonding and consequently the stability of the complex. Figure 2 illustrates the average number of hydrogen bonds (HBs) formed between LYZ–LYZ, LYZ–PAA, and PAA–PAA pairs under varying pH and temperature conditions, while Table 3 includes the numerical values for HBs for all molecular pairs in the system. The hydrogen bonds were calculated by applying a donor–acceptor distance cutoff of 0.35 nm and a hydrogen-donor–acceptor angle cutoff of 30°, following the standard geometric criteria for hydrogen bonding.^60^ We divided the average number of hydrogen bonds in the lysozyme–lysozyme ([LYZ–LYZ]), lysozyme–water ([LYZ–W]), and lysozyme–poly­(acrylic acid) ([LYZ–PAA]) systems with the number of lysozyme protein molecules (16), and in the polymer–polymer ([PAA–PAA]) and polymer–water ([PAA–W]) systems with the number of PAA polymer chains (8).

**Table 3:** Average Number of Hydrogen Bonds (HBs) Formed between Lysozyme–Lysozyme ([LYZ–LYZ]), Lysozyme–Water ([LYZ–W]), and Lysozyme–PAA ([LYZ–PAA]) Shown per Lysozyme Molecule, and PAA–PAA ([PAA–PAA]), PAA–Water ([PAA–W]) Shown per PAA Polymer Chain, Over the Last 100 ns of the Trajectory

Condition	[LYZ–LYZ]	[LYZ–W]	[LYZ–PAA]	[PAA–PAA]	[PAA–W]
pH = 7, *T* = 298 K	102.5 ± 1.2	259.7 ± 2.2	11.6 ± 0.5	1.4 ± 0.3	108.1 ± 2.4
pH = 7, *T* = 330 K	99.2 ± 1.2	250.6 ± 2.2	13.0 ± 0.6	1.3 ± 0.3	100.2 ± 2.4
pH = 7, *T* = 368 K	98.1 ± 1.3	231.1 ± 2.4	14.1 ± 0.7	1.3 ± 0.3	86.8 ± 2.5
pH = 10, *T* = 298 K	97.9 ± 1.3	263.4 ± 2.3	10.7 ± 0.5	0.8 ± 0.3	108.0 ± 2.3
pH = 10, *T* = 330 K	97.1 ± 1.3	249.6 ± 2.3	11.0 ± 0.6	1.2 ± 0.4	99.4 ± 3.0
pH = 10, *T* = 368 K	95.8 ± 1.4	230.1 ± 3.1	12.7 ± 0.6	1.1 ± 0.3	81.3 ± 2.2
pH = 12, *T* = 298 K	99.2 ± 1.2	268.6 ± 2.4	7.6 ± 0.4	1.4 ± 0.3	109.5 ± 2.6
pH = 12, *T* = 330 K	99.3 ± 1.3	249.2 ± 2.4	8.9 ± 0.6	1.8 ± 0.4	99.4 ± 2.6
pH = 12, *T* = 368 K	95.8 ± 1.6	232.3 ± 3.2	10.1 ± 0.6	1.8 ± 0.4	81.5 ± 2.7

**Figure 2:**
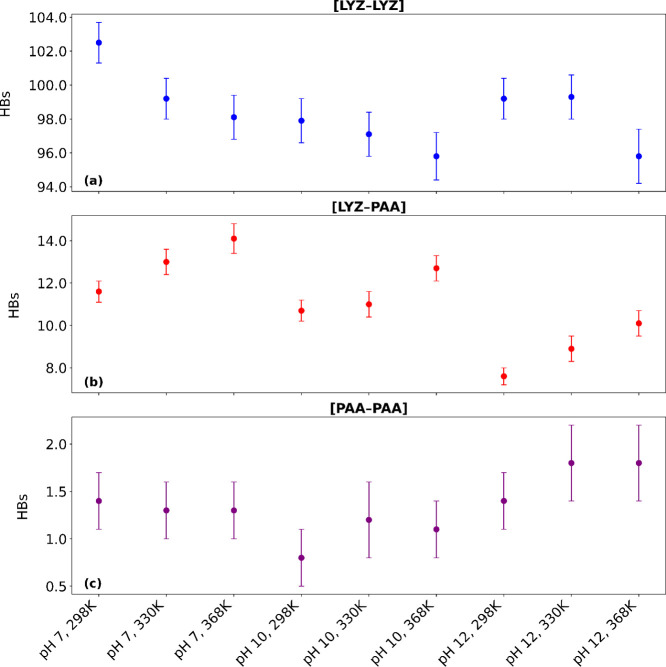
Average number of HBs measured during the last 100 ns of the simulations. (a) the number of [LYZ–LYZ] HBs per lysozyme molecule, (b) [LYZ–PAA] HBs per lysozyme molecule, and (c) [PAA–PAA] HBs per polymer chain, under varying pH and temperature conditions.

At pH 7, the number of hydrogen bonds between LYZ–LYZ decreases slightly with increasing temperature, indicating that temperature does not have a major effect on the intraprotein interactions. In contrast, the hydrogen bonds between LYZ and PAA increase with temperature, suggesting an increase in protein–polymer interaction strength at higher temperatures, which is in line with the findings of Δ*G*_binding_.

Additionally, we observed a slight decrease in hydrogen bond interactions between LYZ and water with increasing temperature, with a similar trend in PAA–water interactions. This is due to the increased level of hydrogen bonding between LYZ and PAA molecules. As the temperature rises, the system undergoes changes in interaction patterns, reducing the available hydrogen bonding sites originally accessible to water molecules. Moreover, PAA–PAA interactions remain relatively stable across the temperatures. Increasing the pH to 10, hydrogen bonds follow a similar pattern for all the pairs but the PAA–PAA, where slightly reduced hydrogen bonds are found at 298 K. At pH 12, the temperature effect on protein–protein hydrogen bonding is observed beyond 330 K, with a decreased value at the highest temperature. The LYZ–PAA hydrogen bonds increase, whereas both LYZ–water and PAA–water hydrogen bonds decrease, similar to the other pH levels. However, for PAA–PAA, increased hydrogen bonding with increasing temperature is observed. Comparing hydrogen bonding between the protein and polymer, the number of hydrogen bonds between LYZ and PAA diminishes as the pH increases from 7 to 10, with a further significant reduction at pH 12, due to the increased negative charge on the protein at higher pH, which repels the similarly charged PAA polymer.

A graphical representation of the variations in hydrogen bonding among LYZ and PAA molecules is presented in Figure 2. At all pH levels, the number of hydrogen bonds between LYZ–LYZ decreases with increasing temperature, whereas a simultaneous increase in LYZ–PAA hydrogen bonding is observed. Temperature has a minimal effect on the number of PAA–PAA hydrogen bonds across all pH values.

In summary, while elevated temperatures strengthen LYZ–PAA hydrogen bonds, increasing pH weakens them. Moreover, while temperature changes have little impact on PAA–PAA interactions, pH variations result in a greater number of PAA–PAA hydrogen bonds at the highest pH value.

#### 3.1.3 Radial Distribution Functions (*g*(*r*))

To investigate protein–polymer relative arrangement, as a result of the aforementioned interactions, we calculated the center of mass pair radial distribution function, *g*(*r*), for the LYZ–PAA complex, focusing on the distribution of PAA chains around the protein at different pH and temperature levels, as shown in Figure 3. In Figure 3b,d,f, with increasing pH, the probability of finding PAA chains around the LYZ protein decreases, which is evident from a reduction in the first peak of the *g*(*r*). This is in agreement with the weakening of protein–polymer binding energy observed as pH values increase, and suggests that the polymer molecules are less likely to be in close proximity to the protein. In contrast, the effect of temperature on the systems, as shown in Figure 3a,c,e, shows that increased molecular motion at higher temperatures increases the likelihood of approach between LYZ and PAA at short distances. Moreover, the slightly broader distribution of PAA around the LYZ protein at higher temperatures indicates more extensive complexes. The pH effect shows consistency across temperature values, while the temperature effect is consistent across the various pH values.

**Figure 3:**
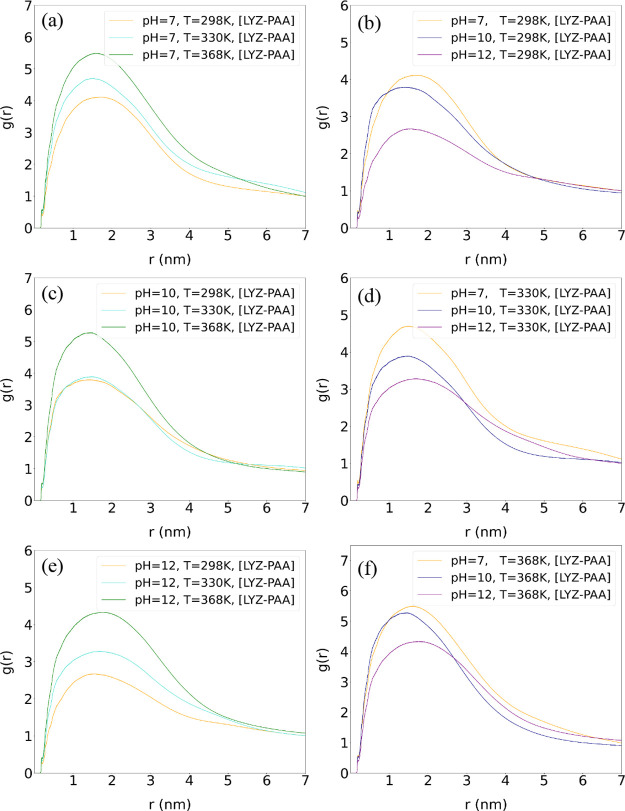
Pair radial distribution functions, *g*(*r*), of the simulated systems over the final 100 ns of the trajectory, calculated between the centers of mass of PAA and LYZ molecules at different temperatures (a, c, e) and at different pH levels (b, d, f).

Next, to explore how pH and temperature affect the ionic condensation around the protein, we probe the pair radial distribution function, *g*(*r*), between the Na^+^ and Cl^–^ ions and the LYZ protein. Data for the ion-LYZ *g*(*r*) are shown in Figure 4 for the systems at different temperatures (a,c,e) and pH (b,d,f) values. The effect of temperature on the distribution of ions around the protein is not particularly strong up to 330 K, whereas ionic condensation is more pronounced at the highest temperature value (368 K) across all pH values. Concerning pH changes, although no significant differences are observed in the *g*(*r*) curves at different pH levels, a slightly higher peak is found at pH 12 at 330 K and more pronounced at 368 K, indicating enhanced ionic condensation.

**Figure 4:**
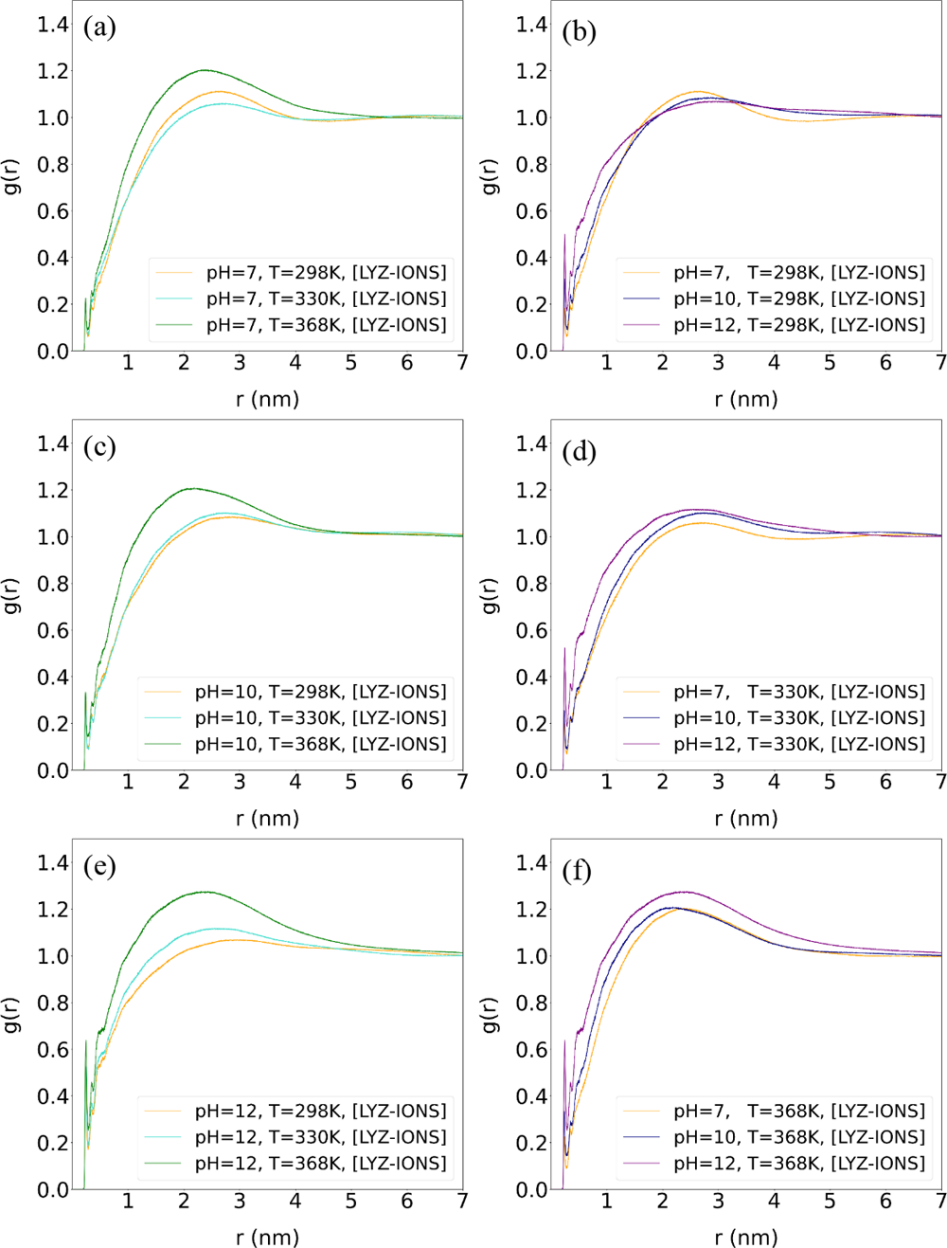
Pair radial distribution functions, *g*(*r*), between ions and LYZ atoms (over the last 100 ns of the trajectory) for systems at different temperatures (a–e) and at different pH levels (b, d, f).

#### 3.1.4 Association Rates

While Δ*G*_binding_, *g*(*r*), and hydrogen bonding analyses have provided valuable insights into pH and temperature effects on protein–polymer interactions, a deeper study at the atomic level is needed to pinpoint specific interaction sites. The Δ*G*_binding_ energy decomposition indicates that Coulombic interactions predominantly drive protein–polymer binding, necessitating the identification of charged amino acid residues that interact with the polymer under different temperature and pH conditions. Figure 5 shows the top 10 residues that have the highest association rates at different temperatures (a, c, and e) and pH levels (b, d, and f). Association rates for all residues are visualized in Figure S4, from which the top 10 residues under each condition were identified and are detailed in Table S4. Figure S5 shows characteristic snapshots from the pH 7, 298 K [LYZ–PAA] system, focusing on ARG128—the residue with the highest association rate (Figure 5a). Each snapshot captures how different ARG128 residues form hydrogen bonds with different PAA chains (ACR137, ACR132, or ACR134), highlighting donor–acceptor atoms between the two molecules and corresponding geometric criteria. Therefore, each snapshot shows a distinct hydrogen bonding pattern.

**Figure 5:**
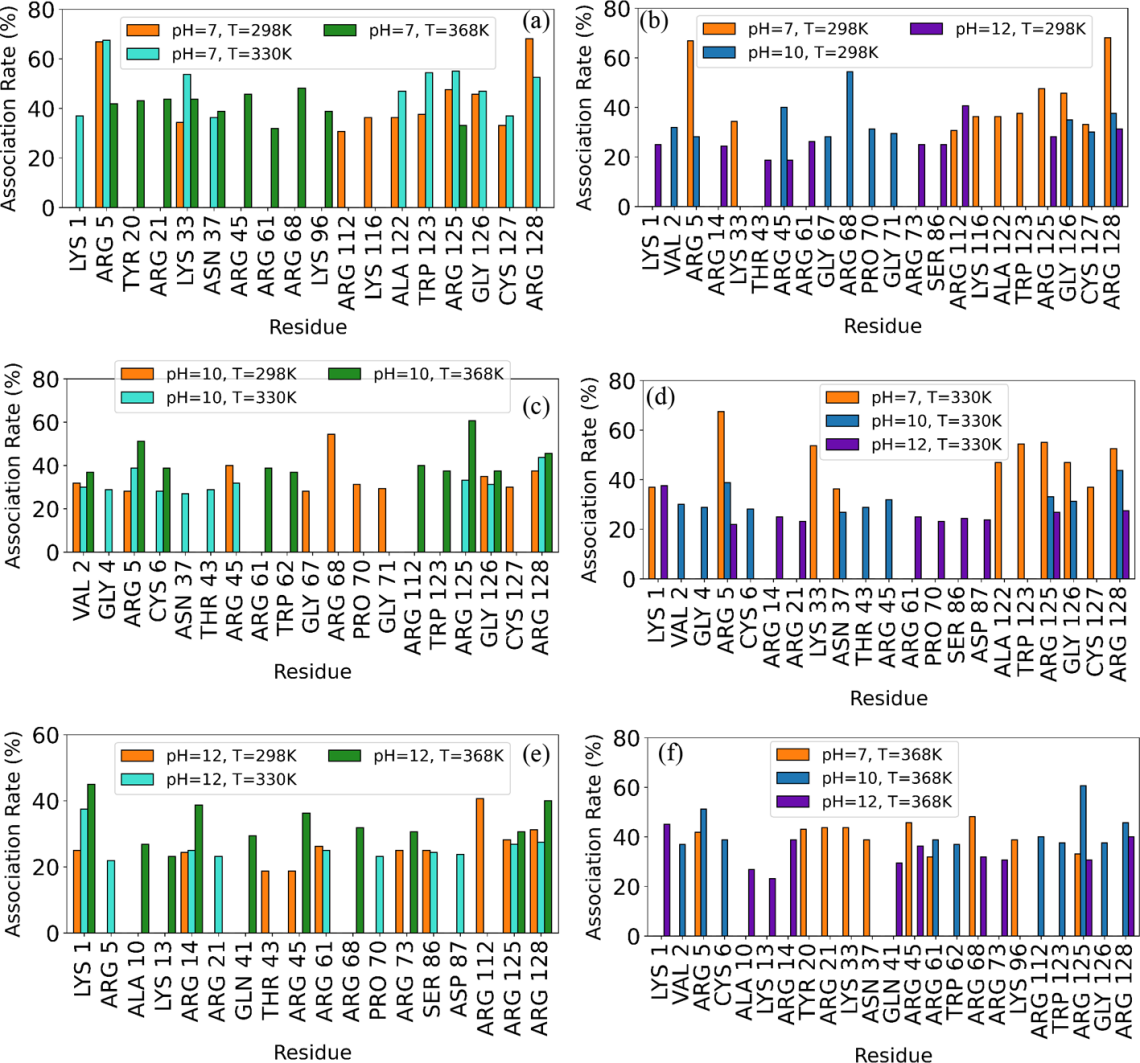
Effect of temperature (a–e) and pH (b–f) on the association rates of the top 10 amino acids with the highest association rates.

To further explore the residue-polymer association, we analyzed the time-wise distances between residue and PAA, for a representative residue (LYS 33) at pH 7 and *T* = 298, 330, and 368 K, as shown in Table S2. This table highlights the time-dependent variability of residue interactions by displaying the fluctuations over the 10 frames captured during the last 100 ns of the trajectory. The variability observed here reflects the dynamic nature of the network formed between the protein and the polymer, and shows how the stability of interactions changes over time. Error bars represent small rearrangements that occur within the complex, which do not alter the overall picture for the association pattern. Since fluctuations over time are minimal in the equilibrated part of the trajectory, indicating stability of the formed network through specific residue-polymer associations, we then focus on environmental variability. Table S3 emphasizes the environmental variability by presenting protein-molecule average distances across the 16 protein molecules for LYS 33 as a representative residue. This analysis reveals how the spatial arrangement of each residue relative to the polymer chains can differ across protein molecules, affecting residue-specific association patterns. Looking at the average distances from the last 10 frames of the simulation in Table S3, we observe differences for the LYS 33 residue across the various protein molecules. This indicates that the spatial arrangement of residue LYS 33 relative to the polymer can vary among different protein molecules. To further quantify these variations, we counted how many times the average distances met the 0.35 nm criterion. The flexibility of the polymer allows it to form bridges between different protein molecules (see Figure 6), which can change the interaction pattern with the proteins, by creating new contact points and altering the local environment around certain residues. These variations among protein molecules highlight the importance of considering both the effect of time and of the local environment in protein–polymer binding.

**Figure 6:**
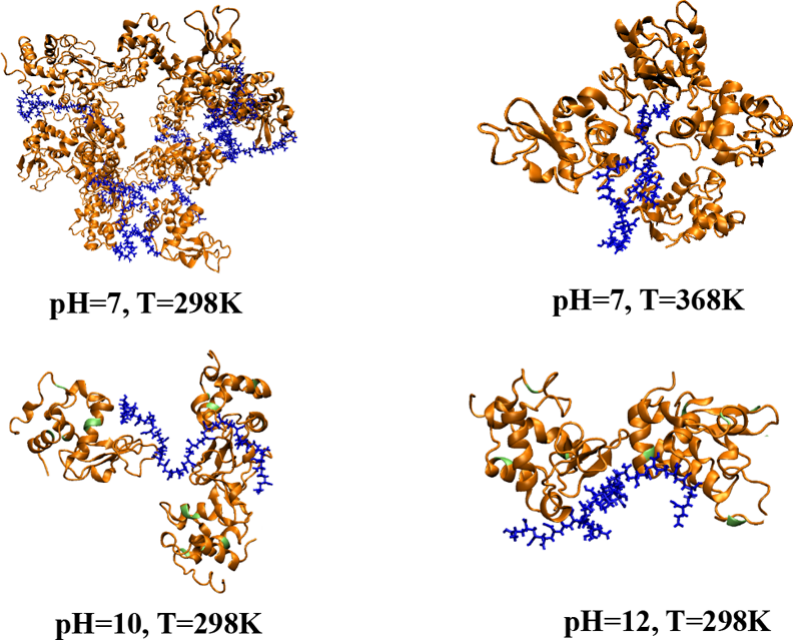
Representative snapshots of protein–polymer bridges in the [LYZ–PAA] complexes at different pH levels and temperatures.

The comparison of the top 10 residues with the highest association rates across pH 7, 10, and 12 at 298, 330, and 368 K reveals clear pH-dependent interaction patterns. At pH 7, strong electrostatic interactions dominate, with arginine (ARG 5, ARG 125, ARG 128) and lysine (LYS 33, LYS 1) consistently exhibiting high association rates across all temperatures due to their stable positive charges. At pH 10, where LYS deprotonation (Figure S1) partially weakens electrostatic forces, a broader distribution of interacting residues emerges, including hydrophobic (VAL 2, PRO 70) and polar residues (ASN 37, GLY 126), indicating increased contributions from van der Waals and hydrogen bonding interactions. However, ARG 125 remains a dominant binder, particularly at 368 K, suggesting a temperature-driven enhancement of binding site exposure. At pH 12, where LYS residues lose their charge (Figure S1), electrostatic contributions diminish, leading to a notable increase in hydrophobic (ALA 10, GLN 41) and polar residues (ASN 19, SER 86) among the top interactors. Despite this shift, ARG, which retains its positive charge in all pH levels, remains a key contributor but with reduced dominance compared to lower pH levels.

Additionally, the effect of protonation states on the polymer interactions with the terminal protein amino acids becomes evident as local changes in charge influence binding patterns. At pH 7, association rates are highest near the N-terminal (ARG 5, ARG 45) and C-terminal (ARG 112, LYS 116, TRP 123, ARG 128) positively charged residues. In contrast, at higher pH levels, deprotonation shifts the dominant interaction regions to residues 61–86, exhibiting increased association rates at pH 10 and pH 12, particularly at 298 and 330 K (Figure S4). Across all pH conditions, temperature amplifies the effect of conformational changes, particularly at 368 K, where hydrophobic interactions become more prominent at higher pH levels.

In more detail, at pH 7, ARG and LYS, in general, dominate across all temperatures, highlighting their stable binding with PAA. In particular, ARG 5 maintains the highest association rate at all temperatures, reflecting its strong binding site proximity. Additionally, glycine (GLY 126) appears among the most associated residues, potentially due to its proximity to charged residues. As the temperature increases from 298 to 368 K, there is a slight redistribution of binding patterns: at 330 K, LYS 33, TRP 123, and ARG 125 gain prominence, indicating temperature-induced conformational changes that expose new binding regions. At 368 K, additional ARG residues (ARG 21, ARG 45, and ARG 68) emerge, suggesting regional thermal destabilization, leading to more exposed binding sites.

At pH 10, the temperature has a more pronounced effect on residue interaction. At 298 K, interactions are dominated by ARG 68, ARG 45, and GLY 126. As the temperature increases to 330 K, the interaction profile diversifies, with several lower-ranked residues, including ASN 39, PHE 3, and GLY 126, appearing among the top 10 residues with the highest association rates. At 368 K, there is a clear dominance of ARG 125, suggesting a temperature-enhanced binding site. Additionally, the emergence of residues such as TRP 123, ARG 112, and ARG 114 shows that the increase in molecular motion allows the polymer to interact with new sites of the protein.

Last, at pH 12, the impact of temperature is most distinct, with LYS deprotonation (Figure S1) reducing electrostatic contributions and leading to more polar and hydrophobic interactions. At 298 K, residues such as ARG 112, ARG 128, and SER 86 dominate. At 330 K, the association rates become more evenly distributed across residues such as LYS 1, PRO 70, and ASP 87 and at 368 K, the dominance of LYS 1 and the appearance of hydrophobic residues, ALA 10, and GLN 41 confirm a clear temperature-driven shift toward van der Waals interactions, as electrostatic contributions diminish with stronger temperature and deprotonation effects.

### 3.2 Effect of pH and Temperature on the Secondary Structure of Lysozyme in the [LYZ–PAA] Complexes

To analyze the structural stability and conformational behavior of LYZ in the [LYZ–PAA] network under different pH and temperature conditions, we computed the root-mean-square deviation (RMSD), the radius of gyration (*R*_g_), and the root-mean-square fluctuation (RMSF) of the protein. The simulation system consisted of 16 independent molecules of LYZ, and the structural properties were computed separately for each LYZ protein and averaged out over all 16 proteins, providing representative values and corresponding deviations for given temperature and pH conditions. Multiple links between LYZ molecules and polymer chains, based on hydrogen bonds or electrostatic interactions, are considered as protein–polymer bridges. These bridges can involve one or more polymer chains and several LYZ molecules, depending on the extent of interaction under different conditions. Representative snapshots of [LYZ–PAA] model complexes, illustrating protein–polymer bridging under different conditions, are shown in Figure 6.

#### 3.2.1 RMSD

The RMSD shown in Figure 7 and Table 4 reveals the combined effect of the temperature and pH on the structural stability of lysozyme within the [LYZ–PAA] complex. The comparison between bulk lysozyme systems ([LYZ]) and lysozyme in complexes (Table 4) demonstrates that the presence of PAA polymer preserves RMSD values of protein similar to its polymer-free state, as reported in our previous study.^32^

**Figure 7:**
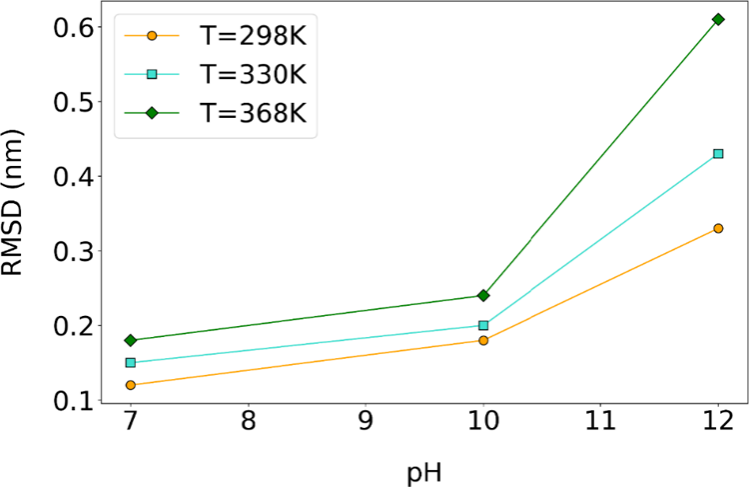
Comparison of average RMSD values for alpha carbons of LYZ over the last 100 ns under different pH and temperature conditions in complexes with PAA.

**Table 4:** Average Alpha Carbon RMSD (nm) Values and *R*_g_ (nm) Values for LYZ at Different pH and Temperatures Over the Last 100 ns of the Trajectory

system	condition	RMSD (nm)	*R*_g_ (nm)
[LYZ]	pH = 7, *T* = 298 K	0.12 ± 0.02	1.38 ± 0.02
[LYZ–PAA]	pH = 7, *T* = 298 K	0.12 ± 0.01	1.45 ± 0.05
[LYZ–PAA]	pH = 7, *T* = 330 K	0.15 ± 0.01	1.45 ± 0.07
[LYZ–PAA]	pH = 7, *T* = 368 K	0.16 ± 0.01	1.46 ± 0.05
[LYZ]	pH = 10, *T* = 298 K	0.18 ± 0.02	1.39 ± 0.16
[LYZ–PAA]	pH = 10, *T* = 298 K	0.18 ± 0.02	1.52 ± 0.11
[LYZ–PAA]	pH = 10, *T* = 330 K	0.24 ± 0.02	1.50 ± 0.10
[LYZ–PAA]	pH = 10, *T* = 368 K	0.28 ± 0.02	1.55 ± 0.13
[LYZ]	pH = 12, *T* = 298 K	0.37 ± 0.12	1.43 ± 0.03
[LYZ–PAA]	pH = 12, *T* = 298 K	0.33 ± 0.03	1.54 ± 0.11
[LYZ–PAA]	pH = 12, *T* = 330 K	0.43 ± 0.02	1.55 ± 0.09
[LYZ–PAA]	pH = 12, *T* = 368 K	0.61 ± 0.04	1.59 ± 0.11

The RMSD results reveal a clear destabilization trend in the LYZ in [LYZ–PAA] complexes with an increase in pH, with the most pronounced conformational changes occurring under highly alkaline conditions. Across all pH values, the conformational flexibility of the protein increases with temperature, but the impact of temperature becomes most significant at pH 12, indicating substantial structural rearrangements. Additionally, RMSD increases with pH, with a larger difference observed between pH 10 and pH 12 than between pH 7 and pH 10, highlighting the destabilizing effect of lysine deprotonation (Figure S1) at higher pH levels. Similar pH-dependent destabilization of lysozyme–polyelectrolyte complexes has been reported by Štajner et al., who observed that reduced positive charge at elevated pH levels weakens electrostatic interactions and promotes greater conformational flexibility.^61^ At pH 7 and pH 10, a small increase in the RMSD values is observed as the temperature rises. This behavior is consistent with the results for binding energies, which show that electrostatic contributions dominate and van der Waals interactions at pH 10 help maintain complex integrity despite thermal effects. In contrast, at pH 12, the RMSD increases sharply, indicating significant structural deviations driven by a collapse of electrostatic stabilization. Our findings for binding energy support this observation, showing that Coulombic contributions become repulsive, weakening the polymer’s stabilizing effect. Although van der Waals forces persist, they are insufficient to counteract the loss of electrostatic binding, resulting in greater conformational flexibility. The increase in RMSD with increasing pH further reflects changes in LYZ’s net charge due to lysine deprotonation, which weakens electrostatic interactions with PAA and leads to more transient, less stable contacts within the network. Consequently, the polymer’s stabilizing effect diminishes, and the protein explores a broader conformational space, contributing to the observed increase of RMSD at higher pH and temperature values.

#### 3.2.2 RMSF

The amino acid-specific degree of stability, quantified by RMSF values (Figure 8), provides insights into local flexibility within the [LYZ–PAA] complex under varying temperature (Figure S6a–c) and pH (Figure 8a–c) conditions.

**Figure 8:**
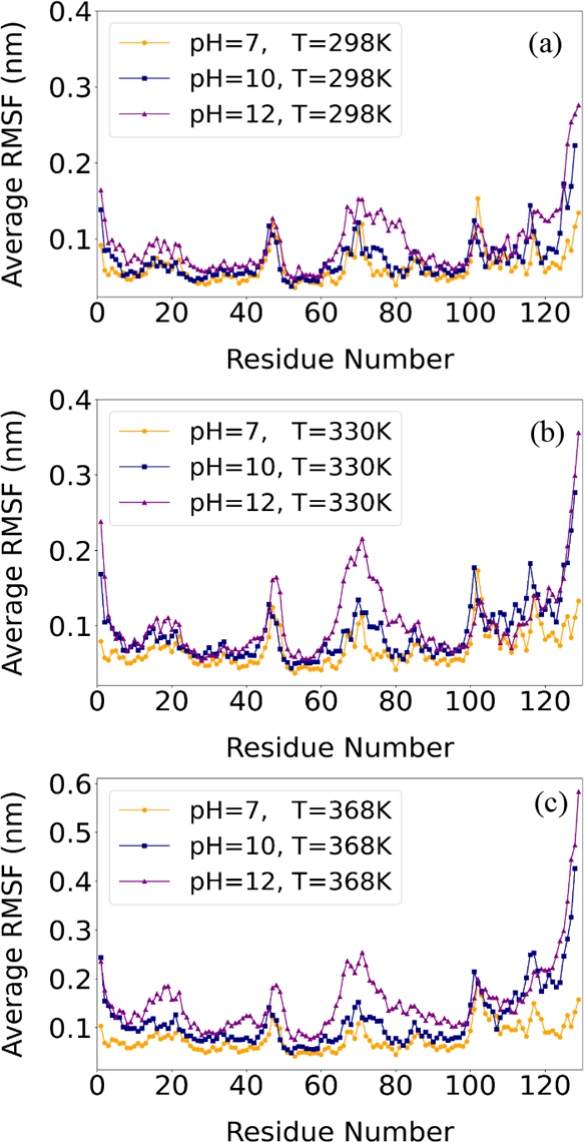
Comparison of RMSF values of backbone atoms of LYZ under different pH levels over the last 100 ns of the trajectory at (a) 298 K; (b) 330 K; (c) 368 K.

While RMSF remains relatively stable between pH 7 and 10, an increase in residue flexibility is observed at pH 12, particularly for residues 61–86 for all temperatures. The increased RMSF in these regions can be attributed to the change of protein–polymer interactions with increasing pH, particularly the shift from electrostatic to hydrophobic interactions, as shown by the association rate analysis and the binding energy calculations. While hydrophobic interactions persist at high pH, they provide weaker stabilization than electrostatic interactions, leading to bigger conformational fluctuations and reduced structural stability. The protein tail (residues >120) is also highly flexible at all pH levels, attributed to increased entropy. RMSF values show minimal changes with temperature (Figure S6a–c), suggesting that local residue flexibility remains relatively small as the temperature rises. Consequently, the presence of the PAA polymer appears to successfully preserve the protein’s secondary structure,^32^ despite extensive overall conformational changes occurring at the highest pH value.

#### 3.2.3 Radius of Gyration (*R*_g_)

The overall size of the LYZ protein, within the [LYZ–PAA] complex, was quantified through the calculation of its value under different temperature and pH conditions. The corresponding results are presented in Table 4. *R*_g_ values remain relatively stable across different temperatures at each pH level, indicating that temperature has a minimal effect on the overall size of the LYZ in complexes, aligning with the moderate changes seen in RMSD. This stability indicates that the polymer effectively preserves the overall structure of LYZ, even in the presence of localized thermal fluctuations, as evidenced by the RMSF at pH 12.

However, the effect of pH on *R*_g_ is more pronounced, showing a clear trend of expansion as the pH increases. At pH 7, the lysozyme in the [LYZ–PAA] complex maintains a relatively compact structure, with an *R*_g_ value slightly larger than that of bulk LYZ, indicating that polymer binding does not significantly alter the protein’s overall size. At pH 10, a noticeable increase in *R*_g_, compared to the corresponding value in the bulk solution, is observed, suggesting that the weakening of electrostatic interactions leads to a more flexible and expanded protein structure, which is consistent with the corresponding RMSD results. A further increase in *R*_g_ is observed at pH 12, with a similar deviation from the corresponding bulk value, indicating substantial expansion. Overall, the increased *R*_g_, along with the high RMSD and RMSF values, supports the conclusion that, at increased pH levels, LYZ adopts a more flexible and expanded conformation within the polymer–protein network. Our findings align with the experimental findings of Morariu et al.,^62^ who observed that pH-dependent conformational changes in lysozyme–polyelectrolyte complexes were associated with variations in zeta potential, suggesting that structural rearrangements influence interfacial electrostatics. In addition, experimental observations by Gummel et al. corroborate this behavior, showing that lysozyme–polyelectrolyte complexes become increasingly expanded at higher pH due to weakened electrostatic interactions arising from reduced protein charge.^63^

### 3.3 Effect of pH and Temperature on the Kinetics of the Complexation Process

In order to explore the effects of pH and temperature on the kinetics of the complexation process of LYZ with PAA, the time evolution of the molecular interactions is monitored. We calculated the number of hydrogen bonds formed between LYZ and PAA, as well as the alpha-carbon contacts between LYZ and PAA over time, providing insight into the dynamic nature of complex formation. The number of contacts between LYZ and PAA was calculated using the GROMACS mindist tool,^64^ defining contacts as α-carbon atoms of LYZ within 0.6 nm of any PAA atom. This analysis shows how molecular rearrangements evolve with time and the effect of the environmental factors on the complexation process of [LYZ–PAA]. Figure 9 shows the change in hydrogen bonding between LYZ and PAA over time at different pH levels, with the hydrogen bonds presented per LYZ molecule. Figure 10 shows the evolution of alpha-carbon contacts between LYZ and PAA over time under different temperatures (a, c, e) and pH conditions (b, d, f). The data for the number of contacts are divided by the total number of LYZ and PAA molecules.

**Figure 9:**
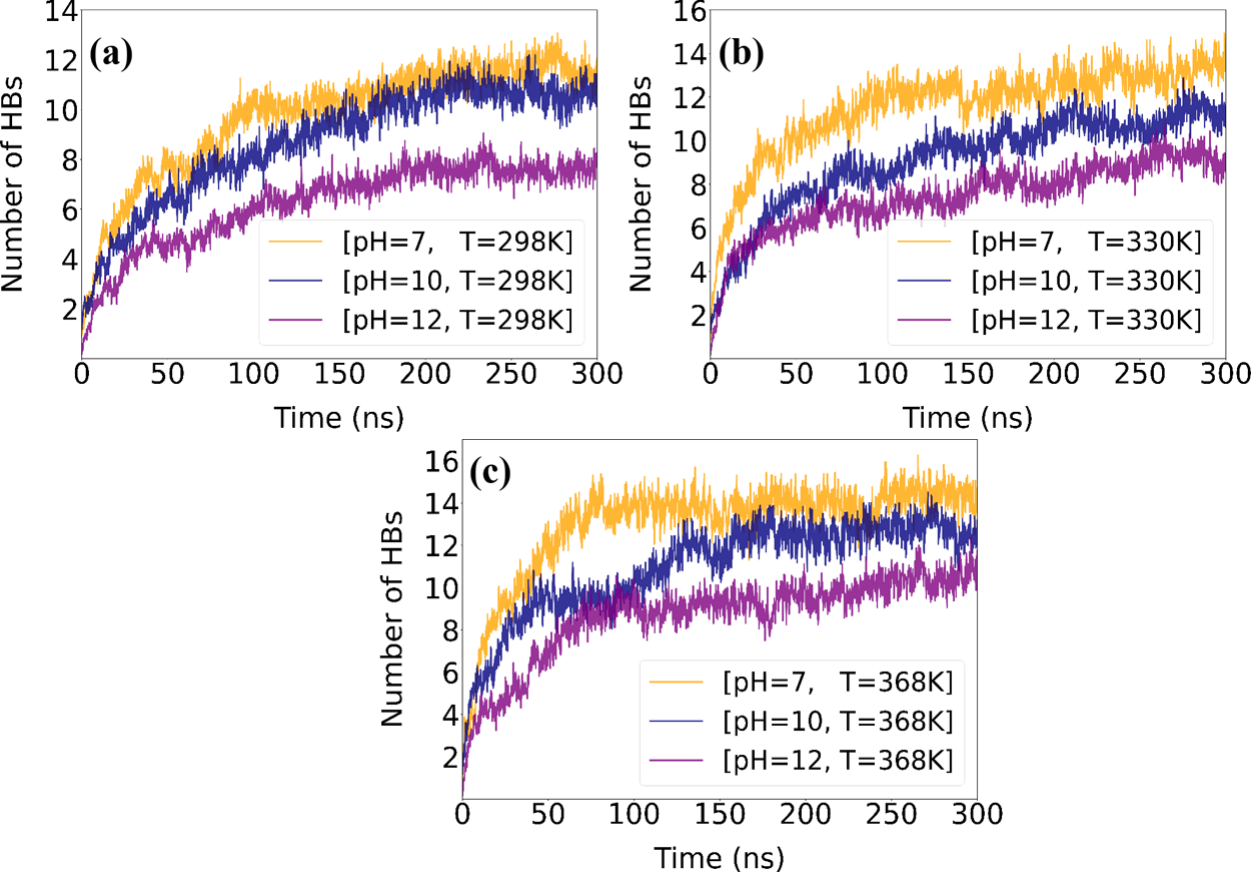
Time evolution of the average number of hydrogen bonds between LYZ and PAA, per LYZ molecule, for different pH (a–c) values.

**Figure 10:**
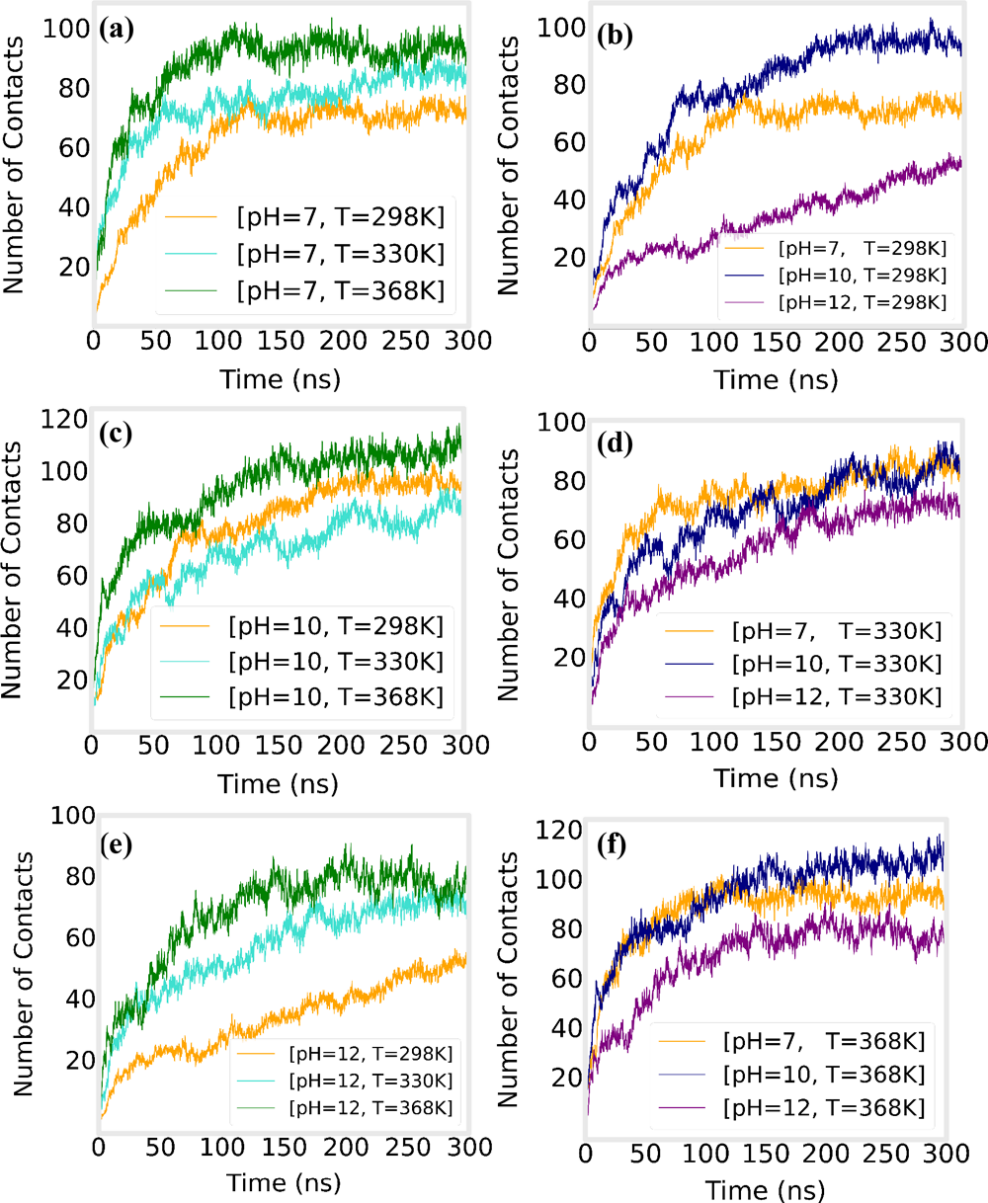
Time evolution of the number of contacts formed between alpha carbons of LYZ and PAA, for different temperatures (a, c, e) and pH (b, d, f) values. Data are normalized with the total number of LYZ molecules and PAA chains.

At pH 7, hydrogen bonding between protein and polyelectrolyte molecules is completed at around ∼100 ns for the highest temperature value (368 K), whereas a gradual retardation is observed at lower temperatures. At 330 K, the plateau is at ∼150 ns, and at 298 K, hydrogen bonds are stabilized at ∼200 ns. Moreover, the completion time of hydrogen bond formation is hardly affected by the pH at all temperatures.

Characteristic times are also extracted from Figure 10, where contact formation seems to be completed at ∼150 ns for all three temperatures at pH 7. In contrast to the hydrogen bonds' time evolution, pH increase has a major impact on how contacts change over time. At pH 10, the plateau is observed around ∼200 ns at all temperature values, and the same characteristic time stands at pH 12 for the two higher temperatures (330 and 368 K). However, at 298 K and pH 12, there is a continuous increase in the number of contacts even up to 300 ns. This indicates that at the highest pH, the molecules within the complex are constantly rearranging, as a consequence of the weakened energetic interactions among them, and at the same time, the slower kinetics at the lowest temperature are apparent. However, although the number of contacts between LYZ and PAA changes continuously at 298 K and pH 12, the number of hydrogen bonds remains constant beyond 200 ns, which suggests that conformational changes do not involve hydrogen bond destruction. Finally, higher temperatures lead to increasing fluctuations in both hydrogen bond formation and Cα contact formation.

## 4 Conclusions

We investigated the effect of the pH and temperature on the complexation process, molecular interactions, and structural stability of [LYZ–PAA] networks using all-atom molecular dynamics simulations. By analyzing binding free energy (Δ*G*_binding_), hydrogen bonds, alpha-carbon (Cα) contacts, association rates, and structural fluctuations, we provided a detailed view of how pH and temperature variation affect protein–polymer interactions and consequently the formed network.

Our results reveal that temperature increase enhances molecular mobility, leading to a more dynamic and transient interaction network between LYZ and PAA. At higher temperatures, fluctuations in hydrogen bonds and Cα contacts slightly increase, indicating a shift towards a more flexible and fluctuating binding interface. Despite these fluctuations, the effect of temperature on Δ*G*_binding_ is relatively small at all pH values, suggesting that temperature-driven kinetic effects do not significantly alter the overall binding affinity between the protein and the polymer. The effect of the pH is more pronounced, primarily influencing electrostatic interactions. As pH increases, binding becomes less favorable (i.e., a reduction in protein–polymer binding strength is observed). This can be attributed to the increased negative charge on LYZ, which leads to electrostatic repulsion with PAA that weakens complex formation. Consistently, the number of hydrogen bonds decreases with increasing pH, further supporting the reduced binding affinity.

The association rate analysis provides deeper insight into these trends, identifying precise interaction sites at the atomic level. Thus, arginine and lysine residues are found to play a dominant role in stabilizing the [LYZ–PAA] complex at lower pH levels, while hydrophobic interactions become more relevant as the pH increases. As the temperature rises, new residues contribute to binding, indicating that thermal fluctuations reveal different interaction sites, leading to dynamic restructuring of protein–polymer contacts. Structural analysis further supports these findings. The overall structure and size of LYZ remain relatively stable at lower temperatures and pH values, while at pH 12, increased flexibility is observed because of weakened electrostatic interactions that allow greater conformational expansion. The RMSF results highlight residue-specific fluctuations, particularly at pH 12 across all temperatures, where a shift from electrostatic to hydrophobic or transient interactions occurs.

The gradual increase in hydrogen bonds and Cα contacts over time indicates a progressive network formation between LYZ and PAA. The kinetics of the [LYZ–PAA] complexation process show distinct patterns depending on the conditions of the aqueous solution. Hydrogen bond formation is largely unaffected by pH and stabilizes within ∼200 ns, with a delay in completion at lower temperatures. However, contact formation is significantly influenced by pH, providing a characteristic time for stabilization within ∼200 ns, for all but the pH 12 and 298 K state, where a continuous increase is observed, suggesting ongoing rearrangements. Despite these rearrangements in contacts of Cα, the number of hydrogen bonds remains stable, indicating that conformational changes do not affect their amount, although they modify the binding pattern.

Overall, our findings demonstrate that temperature enhances molecular mobility, resulting in more fluctuated networks without significantly altering binding strength, while pH has a stronger effect on the interactions and the stability of the complex by modulating electrostatic forces. A key distinction emerges at pH 12, where the negative charge of LYZ (−4) notably reduces its interactions in the aqueous solution when compared with the two lower pH levels. Building on the present findings, we are currently investigating how a specific thermal process, observed in experiments on protein–polyelectrolyte systems, affects the structural integrity, the stability, and the interaction dynamics of LYZ–PAA complexes. This ongoing study is conducted in collaboration with our experimental partners and aims to uncover the development of a given thermal protocol for the stabilization of protein–polyelectrolyte networks. By probing the thermal responsiveness of these complexes, we aim to advance our understanding of their potential for controlled, stimuli-responsive applications in biomaterials. These systems can be engineered to react to environmental triggers, such as pH, temperature, or ionic strength, creating “smart” drug delivery platforms.

## Supplementary Material



## Notes

Supporting Information includes additional data and analyses on the interactions between LYZ and PAA across different pH levels and temperatures (PDF)
