# Supplementary material for: Investigating the Effects of pH and Temperature on the Properties of Lysozyme–Polyacrylic Acid Complexes via Molecular Simulations
Source: ACS Omega. 2025 Jul 31;10(31):34787–800. doi: 10.1021/acsomega.5c03767 (PMC12355258; doi:10.1021/acsomega.5c03767)
Supplement: Supplementary file 1 [file ao5c03767_si_001.pdf]

SUPPORTING INFORMATION

Investigating the Effects of pH and Temperature on the Properties of Lysozyme-Polyacrylic Acid Complexes

Sisem Ektirici[1], Vagelis Harmandaris[1,2,3], Anastassia N. Rissanou[4*],*

1. Computation-Based Science and Technology Research Center, The Cyprus Institute, Nicosia 2121, Cyprus

2. Department of Mathematics and Applied Mathematics, University of Crete, GR-71409 Heraklion, Greece

3. Institute of Applied and Computational Mathematics, Foundation for Research and Technology Hellas, IACM/FORTH, GR-71110 Heraklion, Greece

4. Theoretical & Physical Chemistry Institute, National Hellenic Research Foundation, 48 Vassileos, Constantinou Avenue, GR-11635 Athens, Greece

1. **Structure of Lysozyme at different pH levels**

Understanding the charge distribution of the Lysozyme (LYZ) protein, which varies with the protonation states of its amino acids at different pH levels, is crucial, as it directly influences protein-polymer interactions. Therefore, to clearly illustrate the charge distribution of LYZ at different pH levels and to show which amino acids have been deprotonated, Figure S1 depicts the charge distribution of the protein under various pH conditions. This figure presents a circular representation of the amino acid sequence of LYZ, illustrating residue-specific properties based on their chemical characteristics. Each segment of the circular plot corresponds to one residue in the sequence, ordered from the N-terminal (residue 1) to the C-terminal (residue 129). The color-coding indicates the charge properties of the residues: red for positively charged residues (e.g., Lys, Arg), blue for negatively charged residues (e.g., Asp, Glu), gray for neutral residues and lime for deprotonated residues. Key residues of interest, including deprotonated Lysine (13, 33, 96, and 116) and Histidine (15) residues are highlighted with custom colors and clearly labeled.

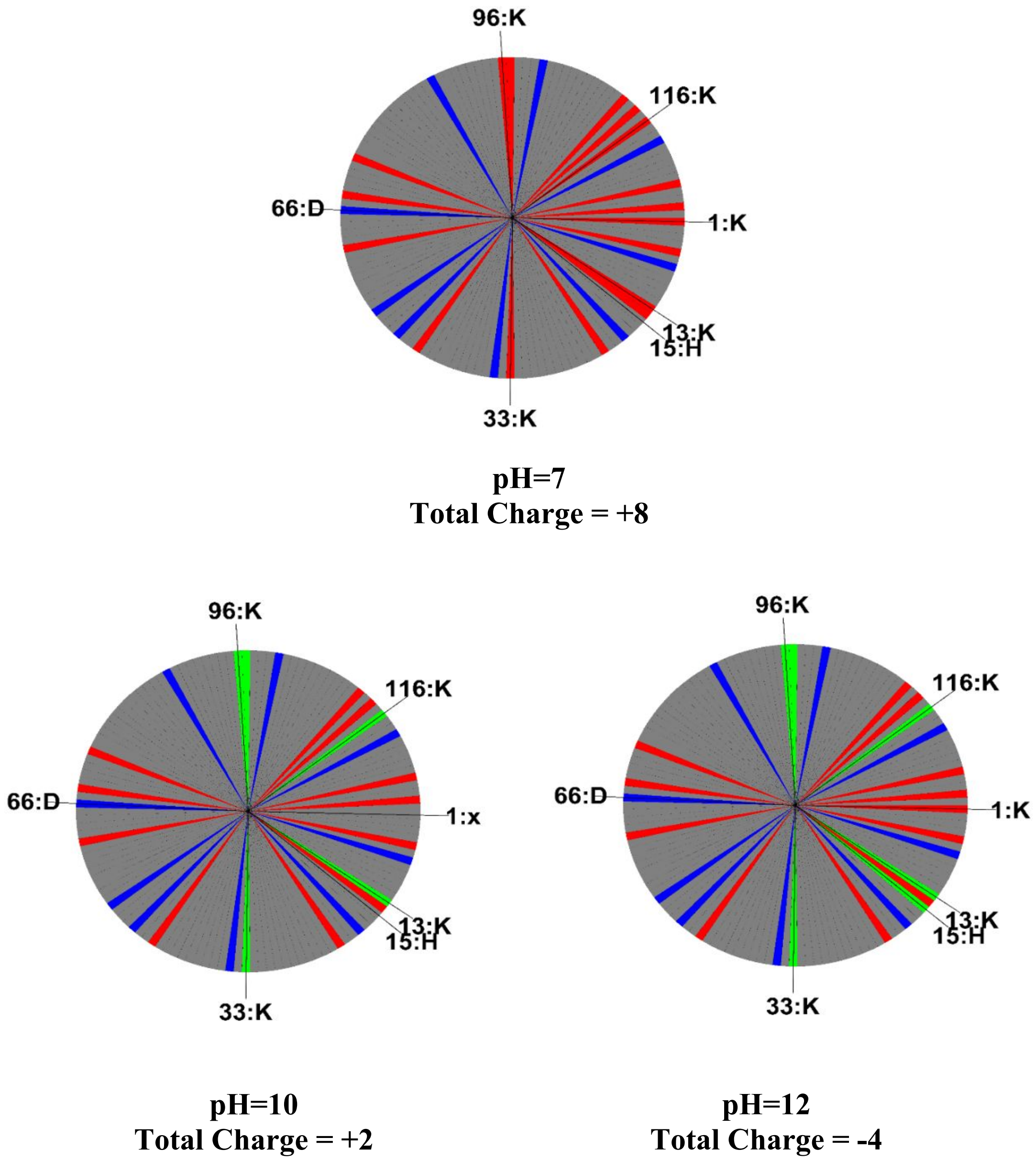

Figure S1. Amino acid and charge distribution of LYZ at different pH levels.

2. Effect of pH and temperature on the [LYZ-PAA] complexation

To comprehensively examine the energy profile in different conditions for the simulated systems, the time evolution of the interaction energy (Figure S2), the binding energy (Gibbs free energy)

and its components (Table S1, Figure S3), and the association rates of the amino acids with PAA polymer (Figure S4) have been calculated.

Total Interaction Energy

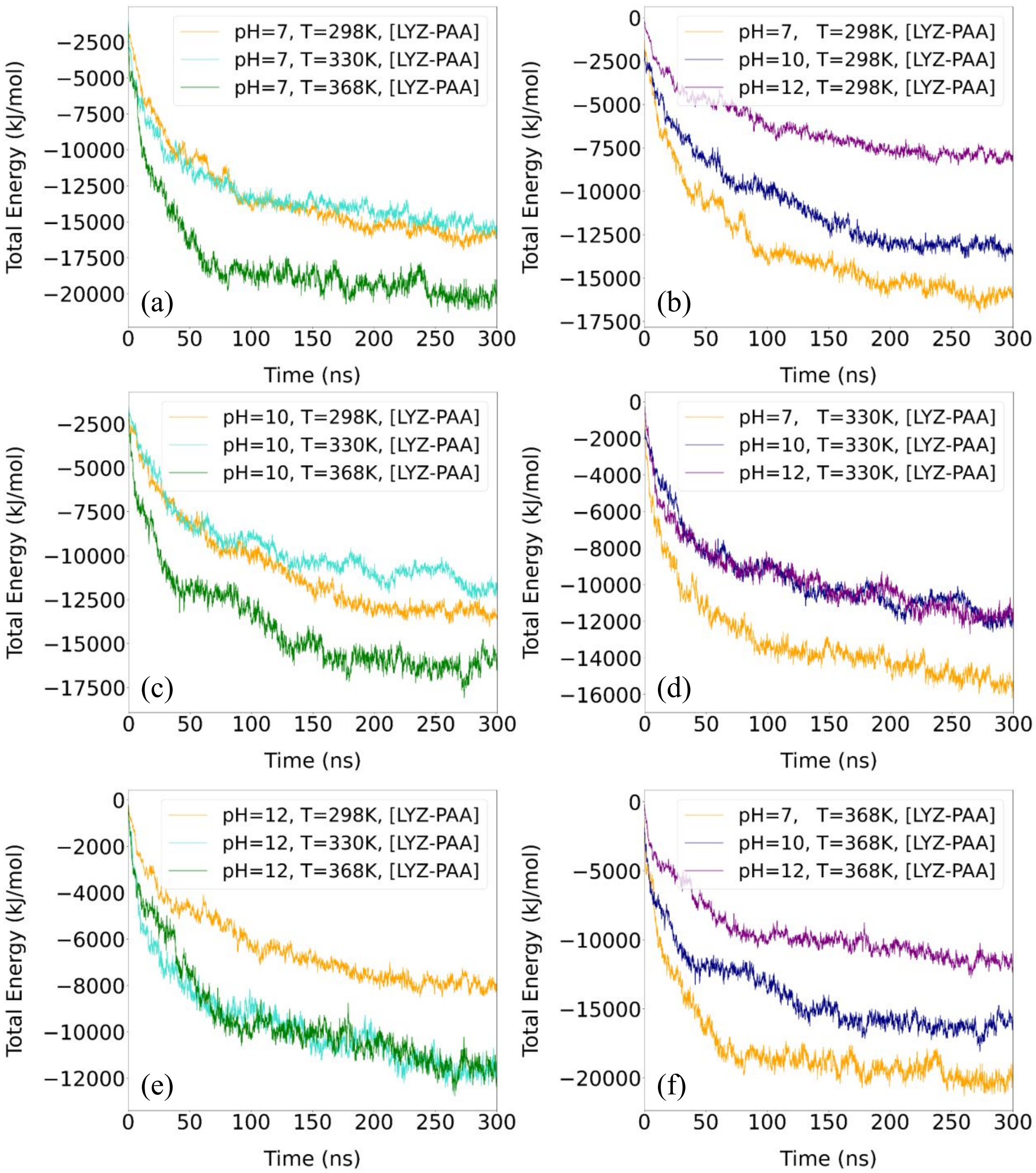

Figure S2. Time evolution of the non-bonded interaction energies between Lysozyme and PAA molecules at different temperatures (a,c,e) and pH levels (b,d,f) throughout the simulation.

Binding Energy

The binding free energy ($\Delta G_{binding}$) was calculated using the gmx_MMPBSA tool, which applies the MM-PBSA (Molecular Mechanics Poisson–Boltzmann Surface Area) method to estimate the free energy difference between the bound complex and its unbound components (LYZ and PAA):

$$\Delta G_{binding} = \Delta G_{complex} - (\Delta G_{LYZ} + \Delta G_{PAA}) \quad \text{Eq. S1}$$

The total free energy (ΔG) for each system was decomposed into gas-phase molecular mechanics or non-bonded energy ($\Delta E_{non\text{-}bonded}$) and solvation free energy ($\Delta G_{solvation}$):

$$\Delta G = \Delta E_{non\text{-}bonded} + \Delta G_{solvation} \quad \text{Eq. S2}$$

Here, the gas-phase non-bonded energy $\Delta E_{non\text{-}bonded}$ accounts for van der Waals ΔE_{VDW} and electrostatic ΔE_{Coul} contributions:

$$\Delta E_{non\text{-}bonded} = \Delta E_{VDW} + \Delta E_{Coul} \quad \text{Eq. S3}$$

The solvation free energy $\Delta G_{solvation}$ consists of a polar component ΔG_{polar} computed using the Generalized Born (GB) method and a nonpolar component $\Delta G_{nonpolar}$ estimated from the solvent-accessible surface area (SASA):

$$\Delta G_{solvation}=\Delta G_{polar}+\Delta G_{nonpolar} \qquad \text{Eq. S4}$$

Thus, the final binding free energy incorporates both the gas-phase molecular mechanics contributions and the solvation free energy differences.

Table S1. Van der Waals (ΔE_{vdw}), Coulombic (ΔE_{Coul}), total non-bonded ($\Delta E_{non\text{-}bonded}$) and binding free energies ($\Delta G_{binding}$) (kJ/mol) of the [LYZ-PAA] complexes at different pH and temperature conditions.

**System**	**Condition**	**ΔE_{vdw}**	**ΔE_{Coul}**	**$\Delta E_{non\text{-}bonded}$**	**$\Delta G_{binding}$**
[LYZ-PAA]	pH=7, T=298K	-2929.2 ± 5.0	-572271.6 ± 272.4	-575200.8 ± 275.3	-10291.8 ± 7.9
[LYZ-PAA]	pH=7, T=330K	-3517.1 ± 6.3	-604867.9 ± 628.9	-608385.4 ± 635.5	-11227.3 ± 13.0
[LYZ-PAA]	pH=7, T=368K	-3693.6 ± 6.3	-584385.1 ± 758.6	-588078.8 ± 762.3	-11161.2 ± 15.5
[LYZ-PAA]	pH=10, T=298K	-2760.2 ± 13.4	-120534.8 ± 235.6	-123294.9 ± 247.7	-3808.7 ± 13.8
[LYZ-PAA]	pH=10, T=330K	-3130.1 ± 6.7	-152212.7 ± 184.5	-155342.7 ± 188.3	-4858.9 ± 9.6
[LYZ-PAA]	pH=10, T=368K	-3688.2 ± 16.3	-145303.6 ± 564.0	-148991.8 ± 578.6	-4870.2 ± 22.2

[LYZ-PAA]	pH=12, T=298K	-1814.6 ± 6.3	191882.0 ± 315.1	190067.8 ± 310.5	673.2 ± 5.4
[LYZ-PAA]	pH=12, T=330K	-2484.9 ± 9.2	202159.2 ± 326.4	199674.3 ± 320.5	322.6 ± 9.2
[LYZ-PAA]	pH=12, T=368K	-2473.2 ± 10.0	181413.6 ± 535.6	178940.9 ± 528.9	581.2 ± 9.2

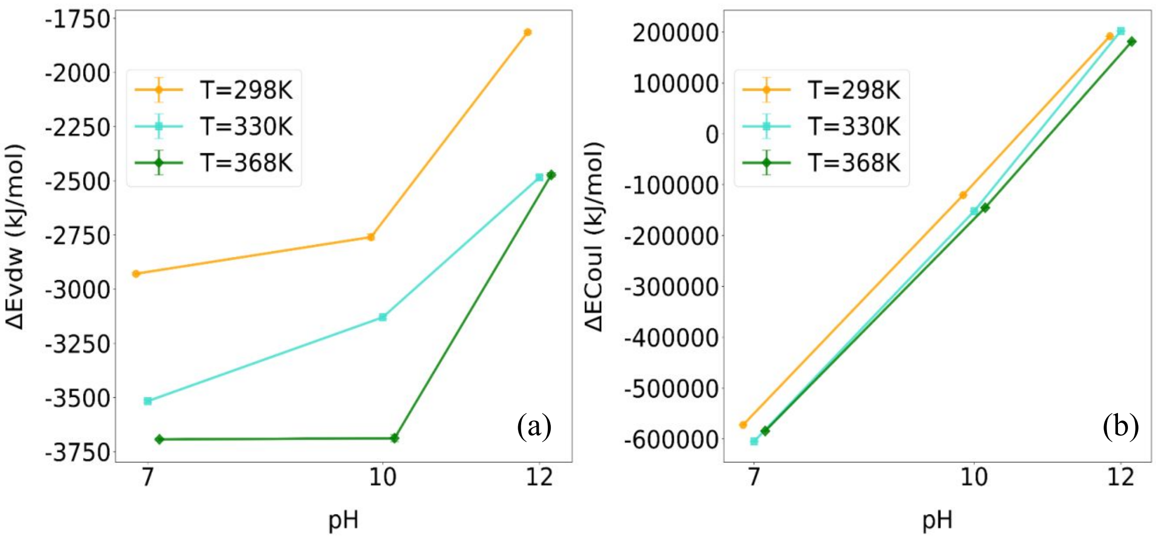

Figure S3. Comparison of (a) ΔE_{vdw} and (b) ΔE_{coul} energy components for [LYZ-PAA] complexes, at the equilibrium state, across various pH and temperature values.

Association Rates

We calculated the association rates to quantify residue-level interactions between LYZ and PAA, providing insight into how temperature and pH influence binding patterns and interaction stability. Tables S2 and S3 provide additional information on the calculation of residue association rates. Table S2 displays time-wise distances for selected residues, capturing time-dependent fluctuations. Table S3 presents protein molecule-wise distances across 16 protein molecules, highlighting environmental variability. Additionally, in Figure S4 histograms compare residue association rates of LYZ with PAA at different temperatures (panels a, c, e) and pH levels (panels b, d, f), calculated

from the last 100 ns of the simulation trajectory for all (129) residues of LYZ. Figure S5 shows a close-up snapshot of the simulation of the most associated residue (ARG128)-polymer chain interaction site. Table S4 shows the names and numbers of the top 10 associated residues under different conditions.

Table S2. Time-wise distances for selected residues, showing time-dependent fluctuations before averaging.

Condition	Residue	200ns	210ns	220ns	…	300ns	$\bar{d}_i$(nm)
pH=7, T=298K	LYS 33	0.38	0.52	0.39	…	0.41	0.35± 0.08
pH=7, T=330K	LYS 33	0.77	0.83	0.75	…	0.20	0.62 ± 0.21
pH=7, T=368K	LYS 33	1.97	1.95	1.99	…	1.91	1.96 ± 0.03

Table S3. Protein molecule-wise average distances across 16 protein molecules for selected residues.

Condition	Residue	PM 1	PM 2	PM 3	…	PM 16	Residues in Chains $\leq$ 0.35	% Association Rate
pH=7, T=298K	LYS 33	0.35± 0.08	3.18 ± 0.16	0.19 ± 0.01	…	1.27 ± 0.16	6	37.5
pH=7, T=330K	LYS 33	0.62 ± 0.21	0.27 ± 0.10	0.19 ± 0.01	…	1.42 ± 0.07	9	56.2
pH=7, T=368K	LYS 33	1.96 ± 0.03	0.19 ± 0.02	0.19 ± 0.02	…	0.19 ± 0.02	7	43.7

Table S4. Top 10 residues with highest association rates across all different pH and temperature conditions.

Condition	Top 10 Residues with Highest Association Rates
pH=7, T=298K	ARG5, ARG128, ARG125, TRP123, GLY126, LYS33, ALA122, CYS127, ARG112, LYS116
pH=7, T=330K	ARG5, LYS33, TRP123, ARG125, ARG128, ASN37, ALA122, GLY126, LYS1, CYS6
pH=7, T=368K	ARG21, ARG45, ARG68, ARG5, TYR20, LYS33, LYS96, ASN37, ARG125, LYS1
pH=10, T=298K	ARG68, ARG45, GLY126, ARG128, VAL2, GLY67, PRO70, GLY71, ARG73, CYS127
pH=10, T=330K	ARG128, ARG5, GLY4, LYS33, ASN39, ARG45, ARG125, GLY126, VAL2, PHE3
pH=10, T=368K	ARG125, ARG5, ARG128, VAL2, PHE3, CYS6, ARG112, ARG114, TRP123, GLY126
pH=12, T=298K	ARG112, ARG128, LYS1, ARG14, ARG61, SER86, ARG125, ASN19, ARG21, GLN41
pH=12, T=330K	LYS1, ARG14, ARG21, PRO70, SER86, ASP87, ARG125, ARG128, ARG5, HISE15
pH=12, T=368K	LYS1, ARG14, ARG45, ARG128, ARG68, ARG125, ALA10, LYS13, GLN41, ARG73

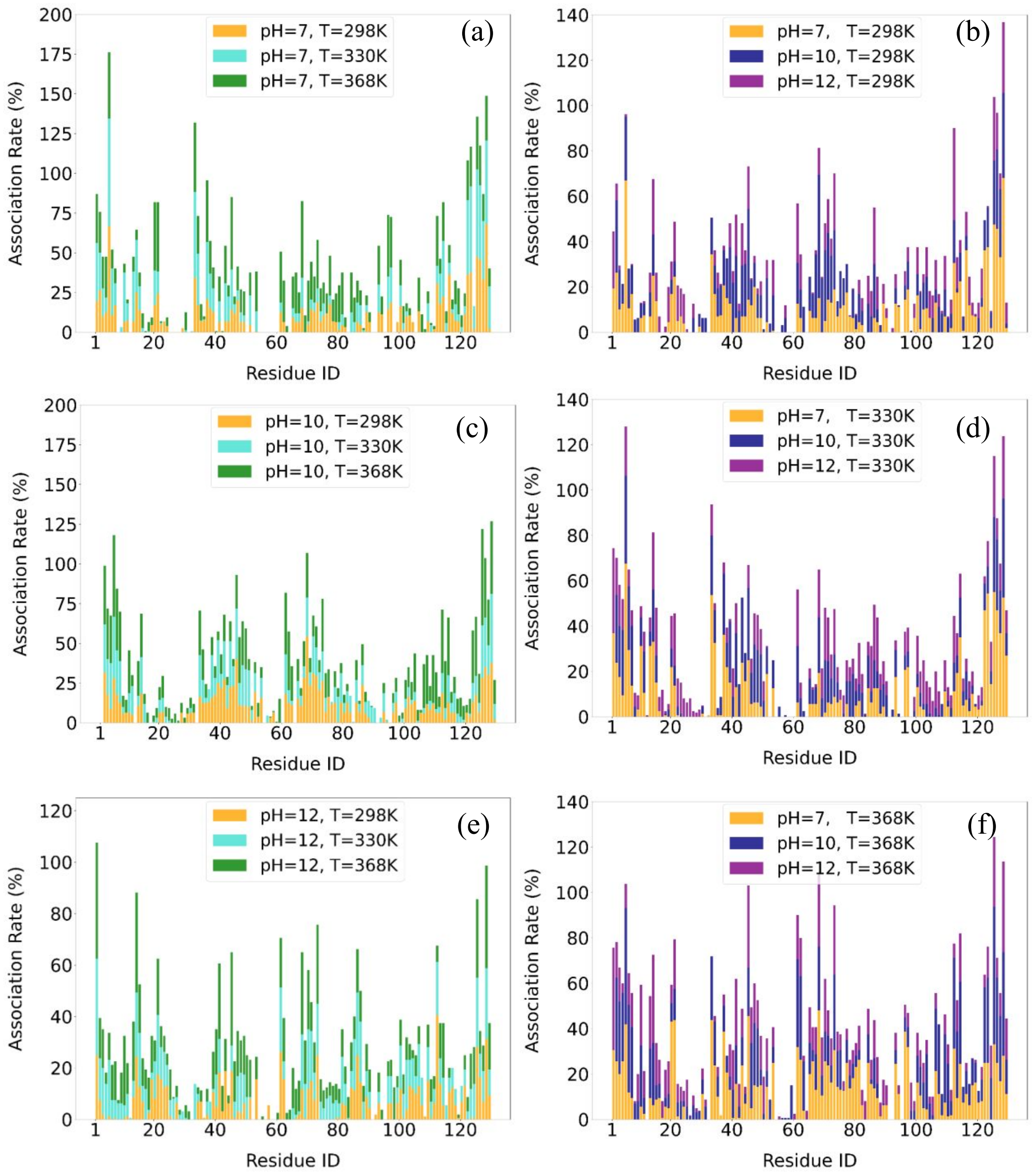

Figure S4. Comparison of residue association rates of LYZ with PAA at different temperatures (a,c,e) and pH levels (b,d,f), represented as histograms calculated from the last 100ns of the trajectory for all (129) residues.

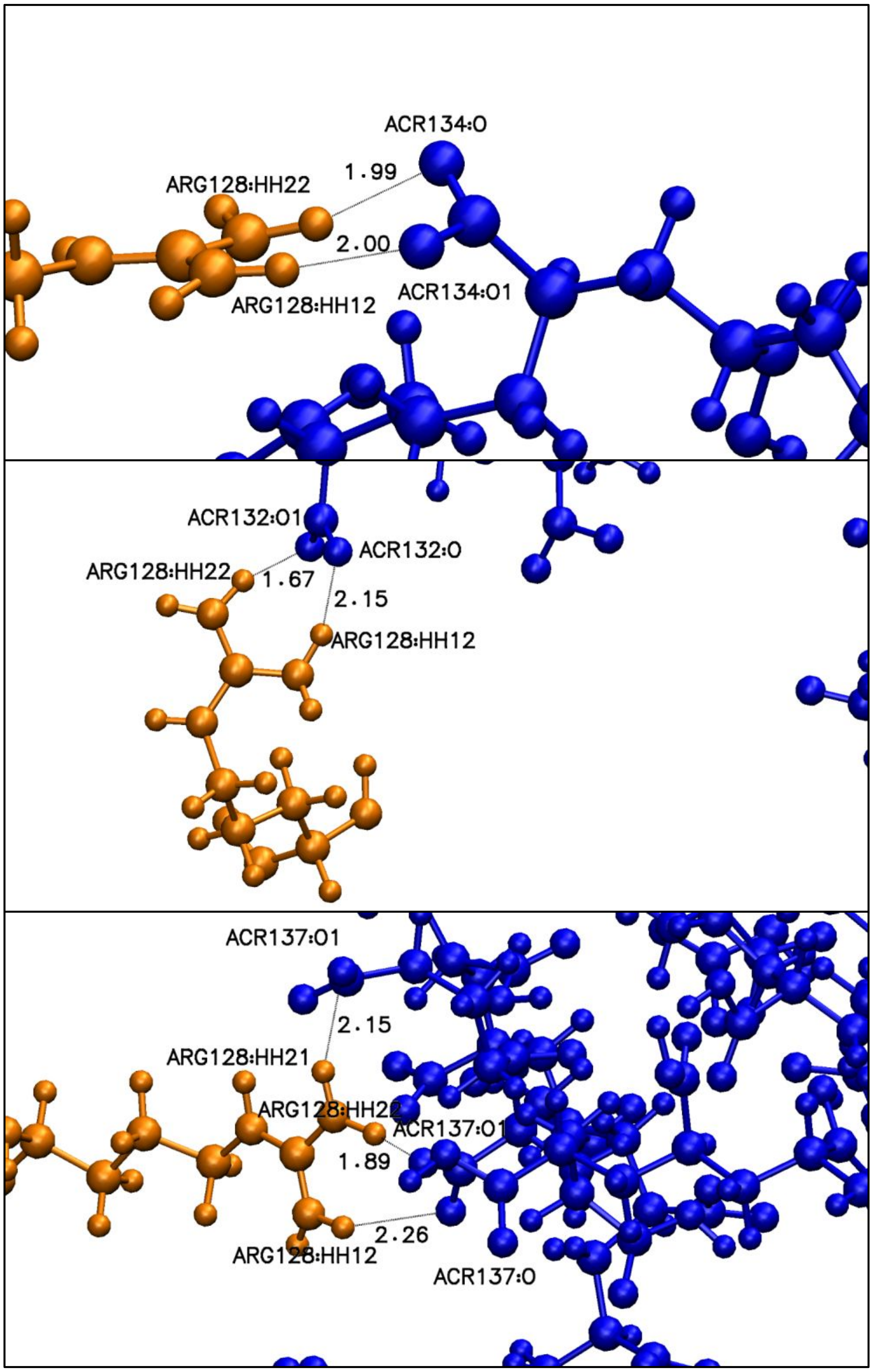

Figure S5. Representative binding sites of the [LYZ–PAA] complex at pH=7, T=298K, showing interaction sites between ARG128 residues of Lysozyme and ACR (PAA) atoms. Hydrogen bond

distances (nm) between ARG128 side-chain hydrogen donors and PAA oxygen acceptors are indicated.

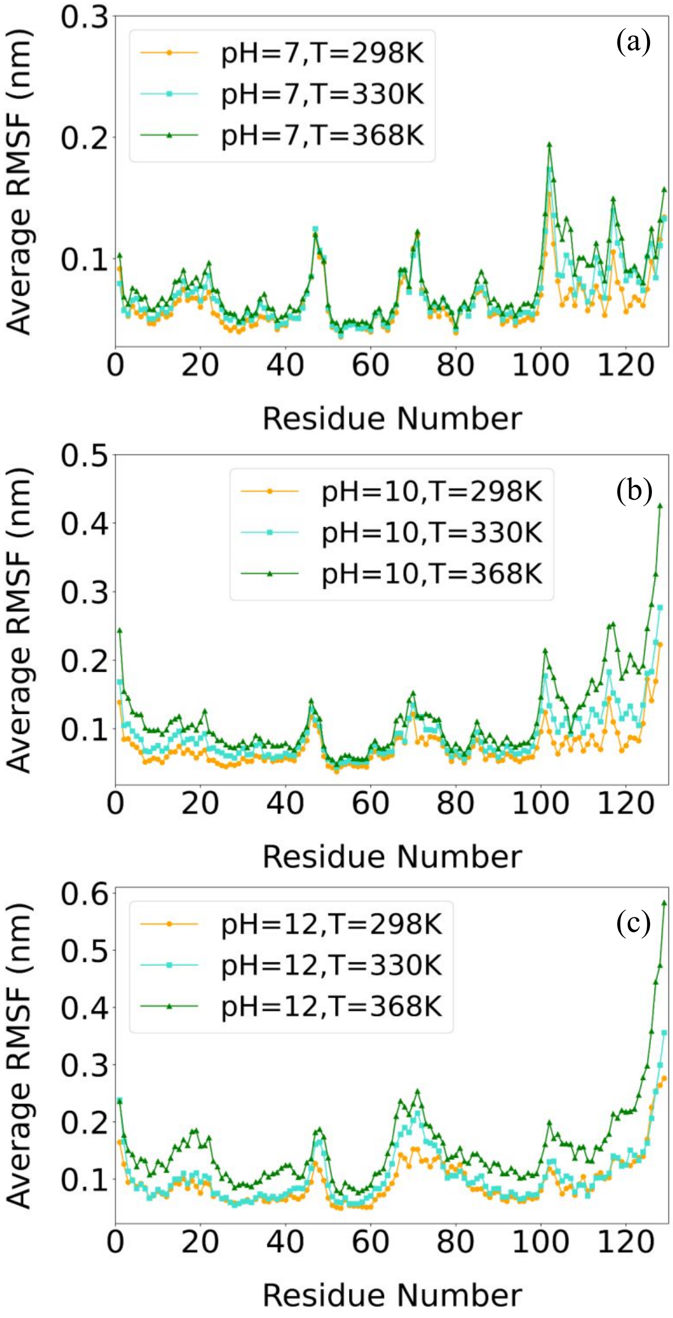

Figure S6. Comparison of RMSF values of back bone atoms of Lysozyme under different temperatures (a,b,c) over the last 100ns of the trajectory.
